# TOURISM study (Treatment Outcomes in UteRIne SarcoMa): a 10-year retrospective evaluation of practice in the UK

**DOI:** 10.1136/bmjopen-2024-094838

**Published:** 2024-12-26

**Authors:** Karen E Mactier, Mark A Baxter, Adam L Peters, Katherine Fair, Laura Hannington, James Robertson, Georgina E Wood, Asma Sarwar, Mai K Bishr, Rebekah Webb, Mohammed Al-Zubaidi, Leonie Eastlake, Katharine Lankester, Samuel McInerney, Helen Creedon, Alison L Stillie, Karin Purshouse, The National Oncology Trainee Collaborative for Healthcare Research

**Affiliations:** 1 Institute of Cancer Sciences, University of Glasgow, Glasgow, UK; 2 Edinburgh Cancer Centre, Western General Hospital, Edinburgh, UK; 3 Ninewells Hospital and Medical School, Dundee, UK; 4 The Beatson Institute for Cancer Research, Glasgow, UK; 5 Beatson West of Scotland Cancer Centre, Glasgow, UK; 6 Ayrshire Hospice, Ayr, South Ayrshire, UK; 7 University College London Hospitals NHS Foundation Trust, London, UK; 8 Radiotherapy, University College Hospital, London, UK; 9 The Institute of Cancer Research, London, UK; 10 Nottingham City Hospital Cancer Care, Nottingham, UK; 11 Royal Derby Hospital, Derby, UK; 12 Weston Park Hospital, Sheffield, UK; 13 Royal Cornwall Hospital, Truro, UK; 14 Royal Sussex County Hospital, Brighton, UK; 15 Institute of Genetics and Cancer, University of Edinburgh Western General Hospital, Edinburgh, UK

**Keywords:** Sarcoma, CHEMOTHERAPY, Gynaecological oncology, Adult radiotherapy

## Abstract

**Abstract:**

**Background:**

Although rare, uterine sarcomas account for a high proportion of uterine cancer mortality. Treatment options and robust trial data are limited.

**Objectives:**

The TOURISM study (Treatment Outcomes in UteRIne SarcoMa) is a UK-wide study by the National Oncology Trainees Collaborative for Healthcare Research which aimed to characterise this patient cohort.

**Design:**

A retrospective descriptive cohort study. Patients with carcinosarcomas/mixed Mullerian tumours, non-uterine gynaecological sarcomas and uterine metastases were excluded. Routine clinical data, including general patient demographics, diagnosis, treatment and outcomes, were collated and pseudonymised.

**Setting:**

Patients diagnosed with uterine sarcoma in the UK National Health Service between 1 January 2008 and 31 December 2017 were identified from electronic records.

**Participants:**

A total of 406 patients from eight centres were eligible for inclusion.

**Results:**

The median age at diagnosis was 56 years, with leiomyosarcoma the most common diagnosis (54.4%). The majority (57.9%) were diagnosed at the International Federation of Gynecology and Obstetrics stage I, with 19.7% diagnosed at stage IV. Nearly half (45.2%) of the patients received at least one line of chemotherapy, of which most (81.0%) received doxorubicin first-line. In the stage I group 7.4% received adjuvant chemotherapy and 15.0% received adjuvant radiotherapy. Median overall survival was 37 months; however, survival varied significantly by stage at diagnosis (stage I: 105 months; stage II: 33 months; stage III: 19 months; stage IV: 14 months).

**Conclusions:**

Our data highlight the diversity in patient management in uterine sarcoma and a marked survival advantage for patients diagnosed with stage I disease. These data highlight the importance of a multidisciplinary approach and describe real-world trends in systemic therapies, radiotherapy and surgical treatment in this rare cancer type.

**STRENGTHS AND LIMITATIONS OF THIS STUDY**

- Large, well-described cohort of a rare condition (n=406).
- Includes up to 10 years of longitudinal follow-up.
- Assessment of multidisciplinary management.
- Trainee clinician-led.

## Introduction

 Uterine sarcomas constitute only 3–7% of uterine malignancies, yet account for a high proportion of uterine cancer deaths.^1^,^3^ Most women are diagnosed in middle age (45–64 years old).^3^ The majority of cases are leiomyosarcomas (LMS) (60%). Other major histological subtypes include endometrial stromal sarcomas (ESS) (25–30%), adenosarcomas (6%) and undifferentiated and other sarcomas (4%).^4^ In common with other anatomical sites, many rarer sarcoma variants may occur, including rhabomyosarcoma and giant cell sarcomas.^5 6^

Histological subtype has a marked impact on prognosis. Survival analysis of 937 patients from the German Cancer Registry diagnosed with stage I/II tumours between 2009 and 2013 found 5-year overall survival (OS) was 53.0% for LMS and 97.2% for low-grade ESS (ESS-LG).^7^ Increasing age and black racial background are also known to adversely affect survival.^8^ Most women present with pelvic pain, bleeding and/or a tumour mass, or are incidentally diagnosed following leiomyoma (fibroid) morcellation surgery.^9 10^ Consequently, many women are initially managed via the gynaecology-oncology rather than sarcoma multidisciplinary team (MDT).

Surgery is the cornerstone of early-stage uterine sarcoma management.^11^ Adjuvant treatment of early disease is hampered by a lack of high-quality evidence from clinical trials. Radiotherapy (RT) may be used with the aim of increasing rates of postoperative local control or to palliate symptoms, but does not improve OS.^12 13^ Similarly, trials of systemic anticancer therapy (SACT), including cytotoxic agents and anti-oestrogen or antiprogesterone agents, have been hampered by small numbers and failed to demonstrate significant survival benefits.^14^ Current UK guidance does not recommend the use of adjuvant SACT or RT.^15^

Doxorubicin-based chemotherapy regimens are the first-line treatment of choice in the metastatic setting, consistent with other soft-tissue sarcomas.^16^ Retrospective analysis of chemotherapy-naïve patients with advanced or metastatic uterine sarcomas treated within the European Organisation for Research and Treatment of Cancer (EORTC) Soft Tisue and Bone Sarcoma Group trials (n=269) failed to demonstrate a significant progression-free survival benefit from treatment intensification to doxorubicin-ifosfomide or cyclophosphamide/vincristine/doxorubicin/dacarbazine regimes compared to doxorubicin monotherapy.^17^ Aromatase inhibitors have been used with some success in ESS.^18^ More recently, targeted agents have been trialled: the VEGF inhibitor pazopanib demonstrated a modest benefit in a small cohort (n=44) of primarily consisting of patients with LMS.^19^ In contrast, the anti-PDGF-Rα monoclonal antibody olaratumab, despite an initial positive phase II study, did not improve outcomes in addition to doxorobicin in a randomised controlled trial.^20^ In summary, uterine sarcomas are a relatively rare, poor-prognosis tumour with limited non-surgical treatment options. Good-quality ‘real-world’ data on treatments received by non-trial populations are lacking. The primary aim of this retrospective analysis is to provide an assessment of current treatment modalities (surgery, RT and SACT) used in UK clinical practice. The secondary aims are to explore the characteristics of this patient group and describe survival outcomes.

## Methods

Women diagnosed with uterine sarcoma between 1 January 2008 and 31 December 2017 were eligible for inclusion in this retrospective descriptive cohort study. Exclusion criteria included histology consistent with carcinosarcomas/mixed Mullerian tumours, non-uterine gynaecological sarcomas and uterine metastases. Input from Sarcoma UK was sought to ensure patient-relevant data points were collected.

UK cancer centres were invited to participate via the National Oncology Trainees Collaborative for Healthcare Research (NOTCH), a UK-wide oncology research collaborative.^21^ Data were collected at each centre by NOTCH representatives. Routine clinical data, including general patient demographics, diagnosis, treatment and outcomes, were hand-searched in the medical records, collated in a Microsoft Excel template and pseudonymised prior to secure electronic transfer to NHS Lothian for central analysis. Overall approval for the completion of this study and information governance procedures was granted by the Lothian NHS Board Caldicott Guardian (application reference 20179). This project was classed as a retrospective service evaluation; therefore, the need for informed patient consent was waived. Caldicott and/or equivalent local clinical governance approvals were obtained in each centre for data collection and transfer.

Analysis was performed in RStudio V.2024.4.2.764 using R V.4.2.2 (2022-10-31 ucrt). Details of additional packages used are presented in online supplemental material. OS was calculated from the date of histopathological diagnosis until the date of death or censor date. Statistical tests, where relevant, are detailed in the text.

In accordance with the journal’s guidelines, we will provide our data for independent analysis by a selected team by the Editorial Team for the purposes of additional data analysis or for the reproducibility of this study in other centres if such is requested, pending consent from the NHS Lothian Caldicott Guardian.

### Patient and public involvement

This was a retrospective review of service delivery, and therefore, the patients included were not directly consulted during the design phase of the project. Advice on the most appropriate patient-centred outcomes was sought from the nurse specialist team at Sarcoma UK.

## Results

Data were collated from 406 women diagnosed with uterine sarcoma from eight UK cancer centres within the 10-year timeframe. Median follow-up was 100.4 months (95% CI 93.1 to 110.3). Baseline clinical and demographic information is presented in online supplemental table 1. The median age at diagnosis was 56 years (range 22–93); the majority (n=240, 64%) of women were postmenopausal. The median number of births per woman was 2 (IQR 0–3; range 0–9). The median body mass index was 29.4 kg/m^2^ (range 17.1–64.3), and most patients were non-smokers (67.1%). Most women were diagnosed at the International Federation of Gynecology and Obstetrics (FIGO) stage I (n=212 patients, 58.0%). LMS was the most common diagnosis at all stages (n=221, 54.5%).

**Figure 1:**
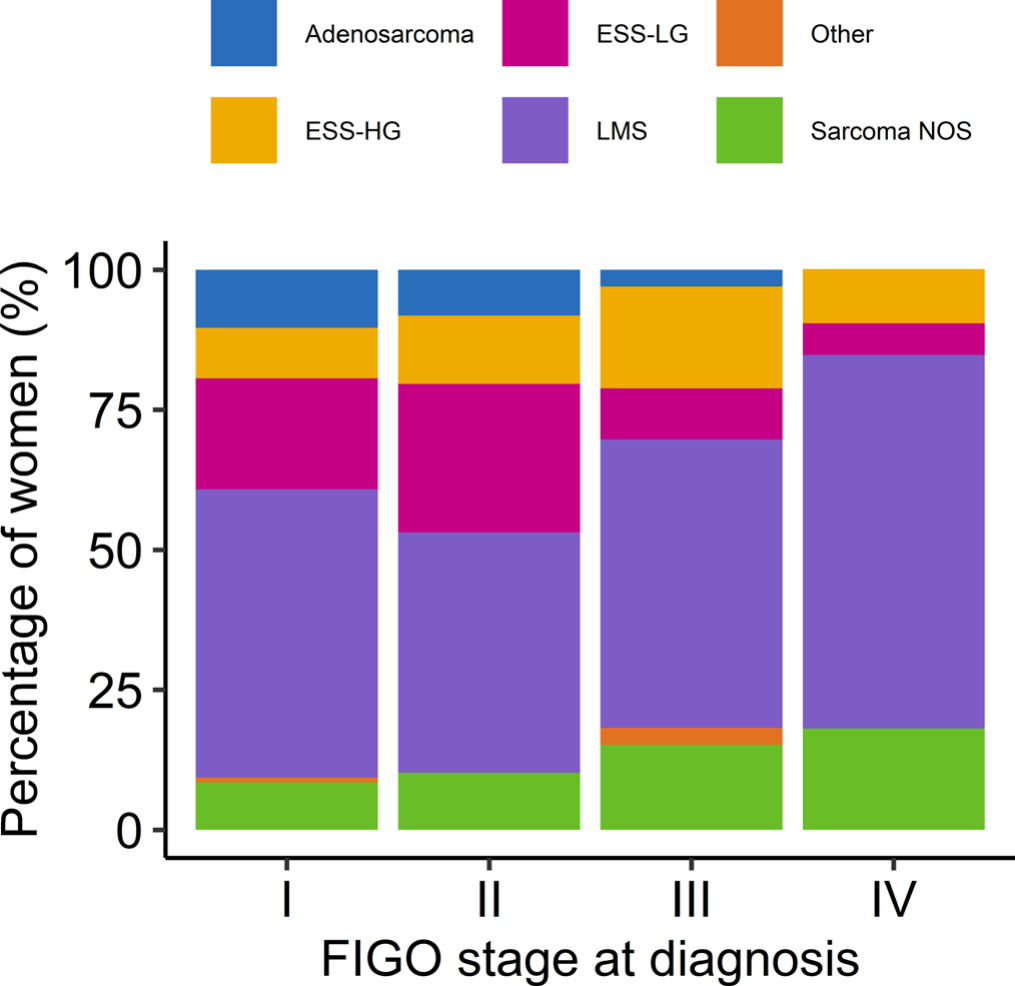
Percentage of women diagnosed at each FIGO stage at diagnosis for each histopathological subtype. ESS-HG, high-grade endometrial stromal tumours; ESS-LG, low-grade endometrial stromal tumours; FIGO, International Federation of Gynecology and Obstetrics; LMS, leiomyosarcomas; NOS, non-specified subtype.

The relationship between FIGO stage at diagnosis and histopathological subtype is presented in figure 1. Patients with adenosarcomas and ESS-LG were most likely to be diagnosed at an early (I–II) stage (96.3% and 88.7%, respectively). This figure was much lower in LMS, high-grade ESS (ESS-HG) and sarcomas of non-specified subtype (NOS) (66.7%, 65.8% and 56.1%).

**Figure 2:**
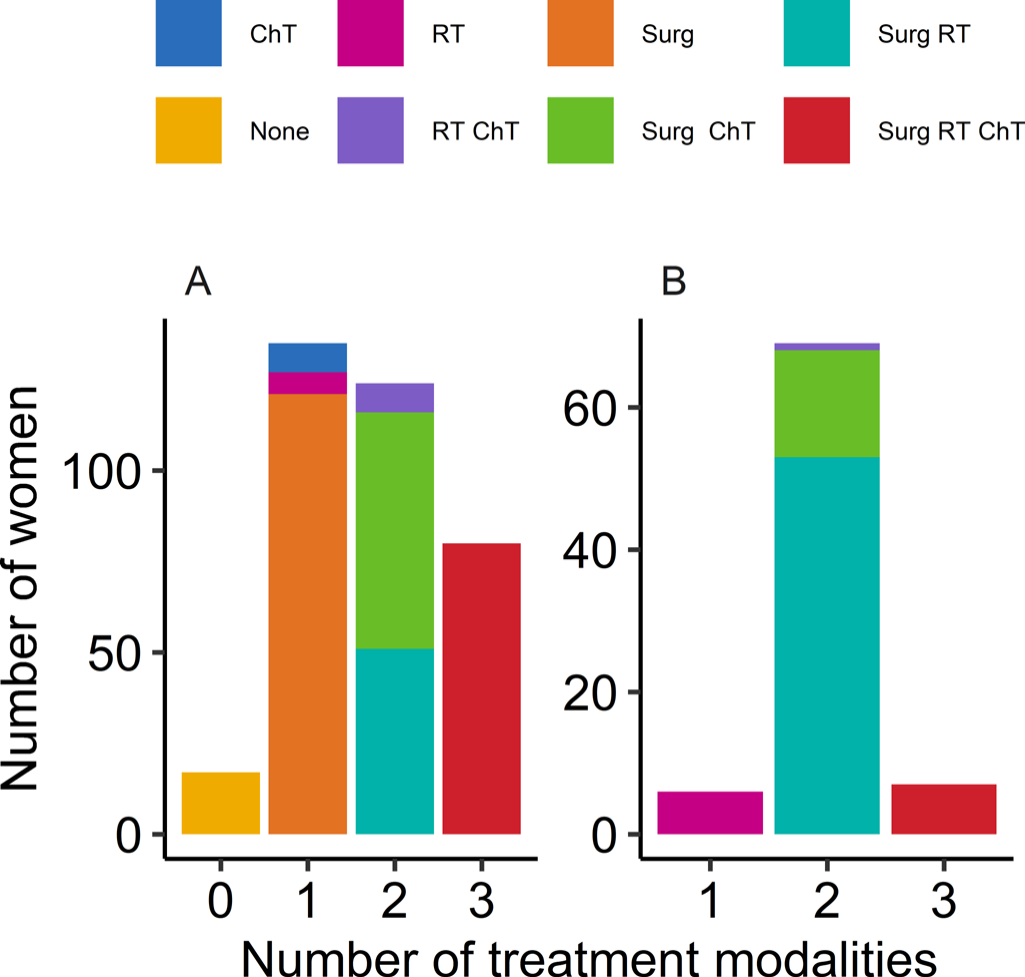
Number of treatment modalities used throughout treatment course. (A) Whole cohort; (B) initial management with curative or adjuvant intent. FIGO, International Federation of Gynecology and Obstetrics. ChT, cytotoxic chemotherapy; RT, radiotherapy; Surg, surgery (excluding biopsy).

Most women received either 1 (37.8%) or 2 (34.8%) modes of treatment over the course of the study period (see figure 2A). Surgery alone (excluding biopsy-only procedures) was the most common treatment option (n=121, 34.0%). Over a quarter (22.5%) had trimodality treatment with surgery, chemotherapy and RT. However, when only treatments given in the curative setting were considered (figure 2B), surgery with adjuvant or salvage RT was the most common treatment pattern (n=53, 64.6%).

Where data were available (n=379), surgery formed part of most women’s management (n=337, 88.9%). Details of surgical procedures by histopathological subtype and FIGO stage are presented in tables1  2, respectively. The most common surgical procedure was an oncological total abdominal hysterectomy and bilateral salphingo-oophorectomy (TAH-BSO) (n=295, 77.8%). Interestingly, 49 (74.2%) patients with stage IVB cancer at diagnosis had surgery, of which the majority (n=39, 59.1%) had a TAH-BSO. Data on indication for surgery were not collected, so it is not known whether these were performed with presumed curative intent before completion of full staging, alongside metastectomy for low-volume secondary disease, or as a palliative procedure for the management of symptomatic local disease.

**Table 1:** Surgical procedures by histopathological diagnosis

	Total (n=406)	Histopathological subtype					
		Adenosarcoma (n=31)	ESS-HG (n=40)	ESS-LG (n=62)	LMS (n=221)	Other (n=6)	Sarcoma NOS (n=46)
Oncological TAH-BSO	295 (78%)	23 (74%)	25 (63%)	52 (84%)	158 (81%)	4 (80%)	33 (72%)
Total hysterectomy	13 (3.4%)	3 (9.7%)	1 (2.5%)	3 (4.8%)	6 (3.1%)	0 (0%)	0 (0%)
Debulking	8 (2.1%)	1 (3.2%)	1 (2.5%)	0 (0%)	4 (2.1%)	0 (0%)	2 (4.3%)
Morcellation	3 (0.8%)	0 (0%)	0 (0%)	1 (1.6%)	2 (1.0%)	0 (0%)	0 (0%)
Other	18 (4.7%)	1 (3.2%)	3 (7.5%)	4 (6.5%)	8 (4.1%)	1 (20%)	1 (2.2%)
No surgery	42 (11%)	3 (9.7%)	10 (25%)	2 (3.2%)	17 (8.7%)	0 (0%)	10 (22%)
Unknown	8 (2.1%)	1 (3.2%)	1 (2.5%)	0 (0%)	4 (2.1%)	0 (0%)	2 (4.3%)

ESS-HGhigh-grade endometrial stromal tumoursESS-LGlow-grade endometrial stromal tumoursLMSleiomyosarcomasNOSnon-specified subtype TAH-BSOtotal abdominal hysterectomy and bilateral salpingo-oopherectomy

**Table 2:** Surgical procedures by FIGO stage

	Total (n=406)	FIGO stage				
		I (n=212)	II (n=49)	III (n=33)	IVA (n=6)	IVB (n=66)
Oncological TAH-BSO	295 (78%)	176 (87%)	42 (88%)	24 (80%)	4 (67%)	39 (60%)
Total hysterectomy	13 (3.4%)	11 (5.4%)	2 (4.2%)	0 (0%)	0 (0%)	0 (0%)
Debulking	8 (2.1%)	1 (0.5%)	1 (2.1%)	0 (0%)	0 (0%)	5 (7.7%)
Morcellation	3 (0.8%)	1 (0.5%)	1 (2.1%)	0 (0%)	0 (0%)	1 (1.5%)
Other	18 (4.7%)	10 (4.9%)	0 (0%)	1 (3.3%)	1 (17%)	4 (6.2%)
No surgery	42 (11%)	4 (2.0%)	2 (4.2%)	5 (17%)	1 (17%)	16 (25%)
Unknown	8 (2.1%)	9 (4.2%)	1 (2.0%)	3 (9.1%)	0 (0%)	1 (1.5%)

FIGOInternational Federation of Gynecology and ObstetricsTAH-BSOtotal abdominal hysterectomy and bilateral salpingo-oopherectomy

Where data were available (n=379), 155 (40.9%) patients had at least one course of RT. RT intent (where recorded) was adjuvant in 57 (36.8%), radical in 9 (15.8%) and palliative in 85 (54.8%) patients. Also, 29 (34.5%) women treated with palliative intent received RT to the primary tumour site alone, 50 (59.5%) received RT to a metastatic site and 5 (6.0%) received RT to both the pelvis and a distal site. No further data on RT dose and symptoms at the time of treatment were available.

**Figure 3:**
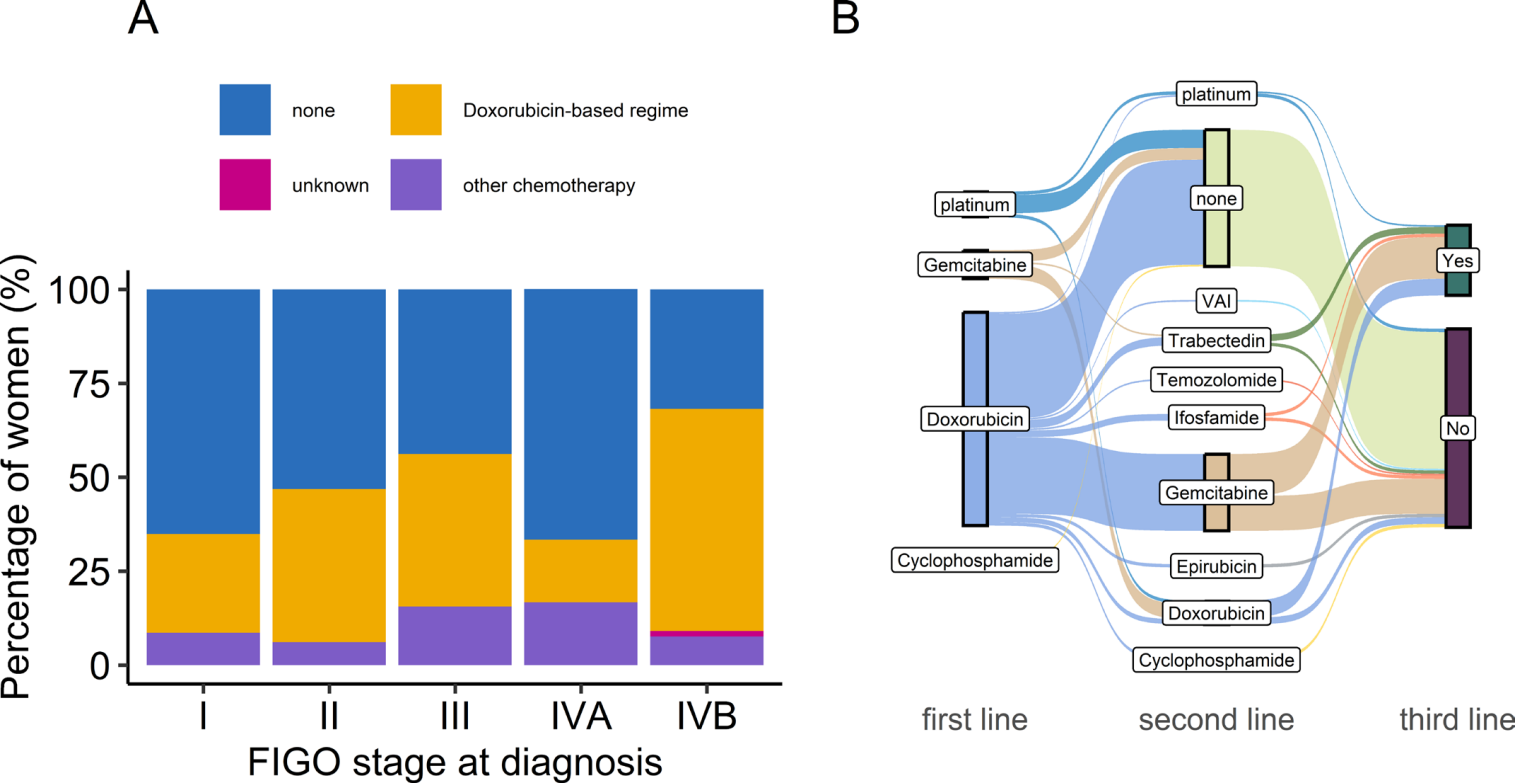
Chemotherapy in the TOURISM cohort. (A) Percentage of women receiving chemotherapy according to the International Federation of Gynecology and Obstetrics stage at diagnosis. (B) Sankey chart demonstrating first- and second-line regime use. Third line yes/no denotes whether third-line chemotherapy was given; type of chemotherapy data was not collected. VAI, vincristine/ actinomycin D/ ifosfamide

Overall, 180 (45.2%) of the cohort received at least one line of cytotoxic chemotherapy. Figure 3 presents (A) the proportion of women receiving chemotherapy by stage at diagnosis and (B) the trends in first and subsequent lines of chemotherapy. The majority (n=146, 81.6%) had a doxorubicin-based regimen in the first-line setting. In total, 17 (9.5%) received a gemcitabine-based regimen (gemcitabine or gemcitabine-docetaxel), 15 (8.4%) had a carboplatin-based regimen (carboplatin single agent, paclitaxel-carboplatin or paclitaxel, etoposide, carboplatin) and one woman (0.6%) received cyclophosphamide. 84 (46.7%) women who had undergone an initial course of chemotherapy went on to receive second-line chemotherapy. Gemcitabine-based regimes were most common in the second line (n=50, 59.5%), followed by doxorubicin-based (n=14, 16.7%). 46 (54.8%) patients who received second-line treatment had at least one more line of therapy (details of third-line chemotherapy were not collected).

The proportion of patients receiving chemotherapy was highest for patients diagnosed at stage FIGO IV (n=47, 65.3%). Over one-third (n=73, 34.9%) of the patients who were initially diagnosed with stage I cancer went on to receive chemotherapy. However, where treatment intent was recorded, it was clear most of these women received chemotherapy for palliation on subsequent disease progression (n=55, 80.9%) while only 13 (19.1%) had received adjuvant chemotherapy.

Hormone treatment was prescribed to 65 (19.6%) women in this cohort (data available: n=331). In total, 35 (10.6%) patients received letrozole, 10 (3.0%) received tamoxifen and 20 (6.0%) received another form of hormone treatment.

**Figure 4:**
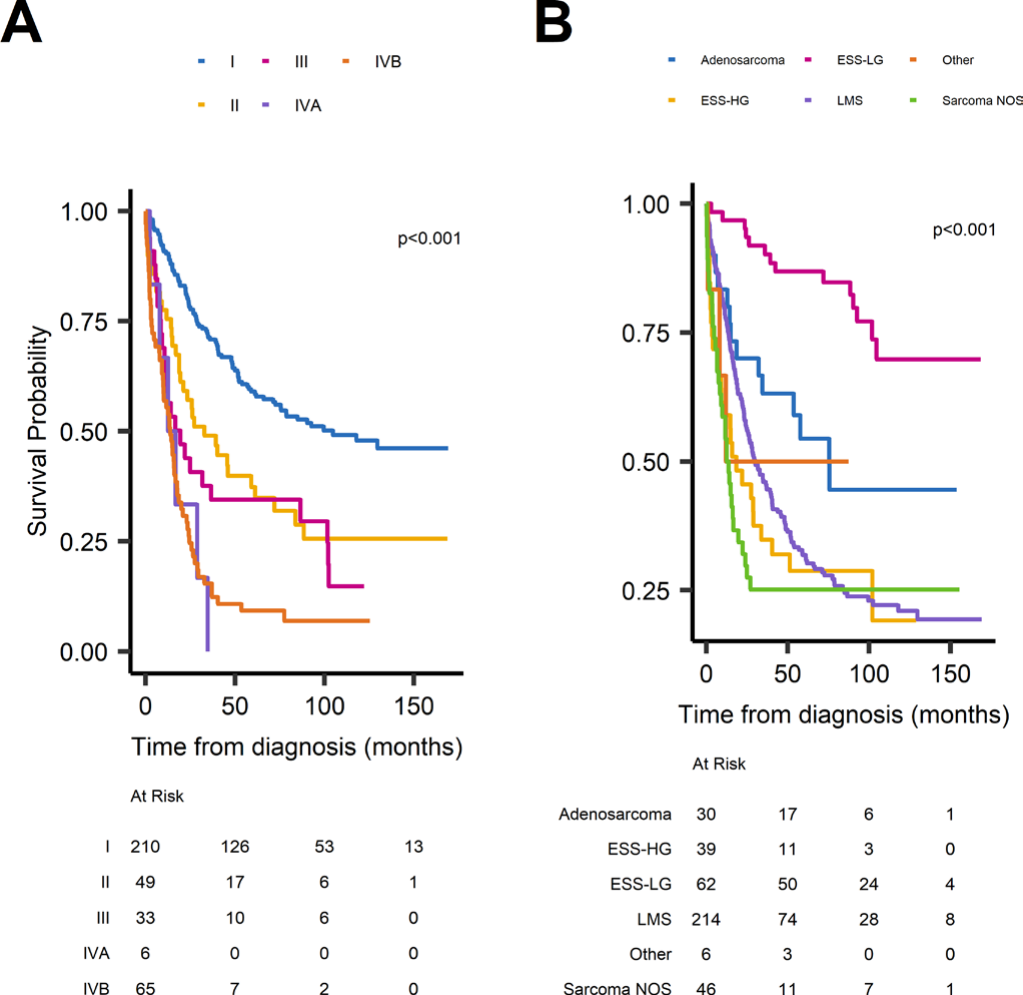
Overall survival (OS). (A) OS by the International Federation of Gynecology and Obstetrics stage at diagnosis. (B) OS by histopathological subtype. P values calculated using the log-rank test. ESS-HG, high-grade endometrial stromal tumours; ESS-LG, low-grade endometrial stromal tumours; LMS, leiomyosarcomas; NOS, non-specified subtype.

Median OS across the whole cohort was 37 months (95% CI 29 to 50), but varied significantly according to stage and tissue diagnosis (figure 4, online supplemental table 2). Median OS was 105 months (95% CI 73 to not reached) in patients diagnosed with stage I disease, falling to 14 months (95% CI 10 to 18) for patients diagnosed with stage IVB. Survival was highest in patients diagnosed with ESS-LG (median OS not reached) but lowest in patients with sarcoma NOS (13.0 months, 95% CI 9.2 to 22). Life expectancy of women younger than the median age (56 years) was almost three times that of older women: median OS 72.0 months (95% CI 48.1 to 104.7) versus 24.1 months (95% CI 18.5 to 32.2).

An analysis of adjuvant therapies in women diagnosed with stage I uterine sarcoma revealed the vast majority of this group had undergone oncological TAH-BSO (n=176, 83.0%, see table 2). In this surgical subgroup, the median OS of patients who had had adjuvant cytotoxic chemotherapy (n=13) was shorter compared with those who did not receive this treatment (n=162), but this trend did not reach statistical significance (78.8 months (95% CI 56.9 to not reached) versus 104.9 months (95% CI 71.4 to not reached), log-rank test: p=0.9). Similarly, median OS was not improved in women who underwent adjuvant RT (n=25; no adjuvant RT: n=142) (median not reached vs 105 months (95% CI 71.4 to not reached), log-rank test: p=0.7). Clinical data justifying or clarifying the decision to use adjuvant treatments in stage I uterine sarcoma were not available, and data on the date of any subsequent disease recurrence within the same subgroup were incomplete. However, we can infer from the prescription of ensuing palliative treatments that at least 67 (38.1%) patients who had an oncological TAH-BSO for stage I uterine sarcoma had recurrent disease, but this is likely to represent an underestimate.

Data was also collected regarding which members of the MDT met with patients. Most patients consulted with both a gynaecologist and an oncologist (n=395, 97.3% and n=351, 88.2%, respectively). In terms of oncology sub-specialisation, 134 (33.0%) met a gynae-specialist oncologist and 171 (42.1%) met a sarcoma-specialist oncologist, while 46 (11.3%) met both a gynae and a sarcoma specialist oncologist.

## Discussion

### Summary of main results

We present the largest retrospective cohort of uterine sarcomas from eight centres across the UK, serving a population of approximately 20 million. Our data demonstrate the highly multidisciplinary nature of uterine sarcoma management and the variation in practice across the UK, particularly after first-line SACT. Although there appeared to be consensus around first-line chemotherapy, with the majority receiving doxorubicin-based treatments, there was far greater variation in the regimen choice in subsequent lines of chemotherapy. As expected, OS was closely related to the stage at diagnosis, with a median OS of 9 years (105 months) in those diagnosed with stage I disease, falling to just over a year in stage IV disease, in agreement with previous reports.^1^

Almost all women with stage I disease received upfront surgery, primarily TAH-BSO. Over 1 in 7 (14.9%) of these patients received adjuvant RT, while approximately half of this number (7.4%) received adjuvant cytotoxic chemotherapy. In stage I in our cohort, there was no significant OS benefit in receiving adjuvant treatment(s) post-TAH-BSO, and a non-statistically significant trend towards poorer survival for those receiving adjuvant chemotherapy. However, numbers were small and did not account for other possible contributory factors (eg, tumour grade, age and physical fitness).

### Results in the context of published literature

The median age and survival data in our study were comparable to another large uterine sarcoma cohort from the German cancer registry, which also found ESS-LG to be the best prognostic group.^7^ UK guidelines estimate LMS comprises approximately 35–40% of all uterine sarcomas.^15^ Our cohort observed a higher-than-expected proportion of patients diagnosed with LMS (55%). Recent guidelines have also highlighted the lack of evidence supporting adjuvant SACT or RT in most cases, which are supported by the findings of our real-world data.^15 22^ Re-analysis of clinical trial data in patients with soft tissue sarcoma of the extremities (percentage LMS: 12–14%) suggested there may be a focused group at high risk of recurrence for whom chemotherapy is beneficial, but this is not currently felt to be the case in uterine sarcomas.^15 23^

Our data also unfortunately confirm the high rate of recurrence in uterine sarcoma, consistent with previous reports in the literature (53–71%).^4^ Our estimate of recurrence in patients with non-stage IVB following TAH-BSO (67.8%) is likely to be an underestimate. UK guidelines recommend a range of chemotherapies for use in the palliative setting, also observed here, including doxorubicin, docetaxel, dacarbazine and trabectedin.^15^ Retrospective evidence suggests Ifosfamide may be less effective in patients with LMS.^24^

Our results also confirm high rates of surgical intervention in patients diagnosed with stage IVB uterine sarcoma. Further work is needed to establish the therapeutic indication for these procedures, especially given concerns regarding the risk of potential intra-abdominal seeding during surgical procedures.^25^

### Strengths and weaknesses

This is a large, multicentre cohort with extensive longitudinal evaluation, encompassing all modalities used in the treatment of uterine sarcoma (surgery, RT, chemotherapy and hormonal management). We also acknowledge the limitations of this study. The retrospective nature means there are incomplete aspects of our data collection. For example, it is likely that hormone treatment, commonly prescribed in primary care, may be underestimated as these data may not be available to TOURISM data collectors. These data can make no inference or claim to the efficacy of different treatment strategies. We appreciate advancements in surgical techniques, for instance, adoption of lymph node dissection, laparoscopy and power morcellation over the study time period may have had a differential effect on outcomes, but was not included in the current work. Our analysis is univariate, and we acknowledge that multiple factors, for which we have not accounted, may impact OS within each of the analysed subgroups.

### Implications for practice and future research

Our study also highlights the truly multidisciplinary nature of treating uterine sarcomas, with most patients receiving more than one treatment modality over the course of their care. The differential survival outcome dependent on age highlights the importance of early diagnosis in a cohort largely represented by postmenopausal women, including access to imaging and tissue biopsy.

Although commonly used, given the high recurrence rate in this cohort, saving doxorubicin-based chemotherapy regimens for the palliative setting may reduce cumulative cardiotoxicity. Clinicians and patients alike would benefit from greater treatment consensus, particularly regarding second-line chemotherapy options. Opportunities for clinicians to share learning and decision-making would be useful for patients and clinicians alike, given the relative rarity of these cancers on a single-centre basis.

## Conclusions

We present, to our knowledge, the largest real-world retrospective analysis of the treatment of uterine sarcoma from multiple centres across the UK. Our data demonstrate that stage at diagnosis is critical to prognosis and highlight the need for early diagnosis. Greater consensus and clarity are required around adjuvant SACT and RT, and optimal second-line chemotherapy options in the metastatic setting.

## supplementary material

10.1136/bmjopen-2024-094838 online supplemental file 1

10.1136/bmjopen-2024-094838 online supplemental file 2

## Data Availability

Data are available upon reasonable request.
