# Supplementary material for: TOURISM study (Treatment Outcomes in UteRIne SarcoMa): a 10-year retrospective evaluation of practice in the UK
Source: BMJ Open. 2024 Dec 26;14(12):e094838. doi: 10.1136/bmjopen-2024-094838 (PMC11683892; doi:10.1136/bmjopen-2024-094838)
Supplement: online supplemental file 1 [file bmjopen-14-12-s001.pdf]

SUPPLEMENTARY MATERIALS

Contributing cancer centres
Beatson West of Scotland Cancer Centre, Glasgow
Sunrise Centre, Royal Cornwall Hospital, Truro
Dundee Cancer Centre, Ninewells Hospital, Dundee
Edinburgh Cancer Centre, Western General Hospital, Edinburgh
Nottingham Cancer Centre, Nottingham University Hospitals NHS Trust, Nottingham
Weston Park Cancer Centre, Weston Park Hospital, Sheffield
Sussex Cancer Centre, Royal Sussex County Hospital, Brighton
University College Hospital Macmillan Cancer Centre, London

Statistical packages

```
R version 4.4.2 (2024-10-31 ucrt)
Platform: x86_64-w64-mingw32/x64
Running under: Windows 11 x64 (build 22631)

Matrix products: default


locale:
[1] LC_COLLATE=English_United Kingdom.utf8
[2] LC_CTYPE=English_United Kingdom.utf8
[3] LC_MONETARY=English_United Kingdom.utf8
[4] LC_NUMERIC=C
[5] LC_TIME=English_United Kingdom.utf8

time zone: Europe/London
tzcode source: internal

attached base packages:
[1] stats     graphics  grDevices utils     datasets  methods
[7] base

other attached packages:
 [1] ggsankey_0.0.99999 glue_1.8.0         snakecase_0.11.1
 [4] gtsummary_2.0.3    ggsurvfit_1.1.0    survminer_0.5.0
 [7] survival_3.7-0     gridExtra_2.3      ggsci_3.2.0
[10] ggpubr_0.6.0       rstatix_0.7.2      janitor_2.2.0
[13] lubridate_1.9.3    forcats_1.0.0      stringr_1.5.1
[16] dplyr_1.1.4        purrr_1.0.2        readr_2.1.5
[19] tidyr_1.3.1        tibble_3.2.1       ggplot2_3.5.1
[22] tidyverse_2.0.0

loaded via a namespace (and not attached):
 [1] tidyselect_1.2.1     farver_2.1.2
 [3] fastmap_1.2.0        broom.helpers_1.17.0
 [5] labelled_2.13.0      digest_0.6.37
 [7] timechange_0.3.0     lifecycle_1.0.4
 [9] magrittr_2.0.3       compiler_4.4.2
[11] rlang_1.1.4          tools_4.4.2
[13] utf8_1.2.4           yaml_2.3.10
[15] gt_0.11.1            data.table_1.16.2
[17] knitr_1.49           ggsignif_0.6.4
[19] labeling_0.4.3       bit_4.5.0
[21] xml2_1.3.6           abind_1.4-8
[23] withr_3.0.2          grid_4.4.2
[25] fansi_1.0.6          xtable_1.8-4
[27] colorspace_2.1-1     scales_1.3.0
[29] cli_3.6.3            rmarkdown_2.29
[31] crayon_1.5.3         generics_0.1.3
[33] rstudioapi_0.17.1    km.ci_0.5-6
[35] tzdb_0.4.0           splines_4.4.2
[37] parallel_4.4.2       survMisc_0.5.6
[39] vctrs_0.6.5          Matrix_1.7-1
```

```
[41] carData_3.0-5       car_3.1-3
[43] hms_1.1.3           patchwork_1.3.0
[45] bit64_4.5.2         Formula_1.2-5
[47] cowplot_1.1.3       stringi_1.8.4
[49] gtable_0.3.6        munsell_0.5.1
[51] pillar_1.9.0        htmltools_0.5.8.1
[53] R6_2.5.1            KMsurv_0.1-5
[55] vroom_1.6.5         evaluate_1.0.1
[57] lattice_0.22-6      haven_2.5.4
[59] backports_1.5.0     cards_0.3.0
[61] broom_1.0.7         cardx_0.2.1
[63] xfun_0.49           zoo_1.8-12
[65] pkgconfig_2.0.3
```

Supplementary data

Characteristic	Total (n = 406)	Beatson (n = 88)	Cornwall (n = 25)	Dundee (n = 15)	Edinburgh (n = 46)	Nottingham (n = 17)	Sheffield (n = 19)	Sussex (n = 33)	UCL (n = 163)	P
**Age at Diagnosis, median (IQR)**	56 (48, 66)	55 (49, 66)	56 (49, 65)	60 (48, 71)	60 (54, 68)	55 (50, 67)	55 (45, 69)	64 (58, 72)	53 (46, 63)	<0.001*
**Menopausal status**										<0.001†
Pre-menopausal	104 (28%)	23 (27%)	4 (17%)	4 (29%)	1 (2.6%)	7 (44%)	5 (26%)	1 (3.6%)	59 (39%)	
peri-menopausal	32 (8.5%)	6 (7.0%)	0 (0%)	1 (7.1%)	3 (7.9%)	0 (0%)	3 (16%)	3 (11%)	16 (11%)	
post-menopausal	240 (64%)	57 (66%)	20 (83%)	9 (64%)	34 (89%)	9 (56%)	11 (58%)	24 (86%)	76 (50%)	
**Smoking status**										0.109†
Current smoker	35 (12%)	17 (22%)	3 (16%)	1 (7.7%)	5 (14%)	2 (25%)	0 (0%)	1 (17%)	6 (4.7%)	
Ex-smoker	62 (21%)	14 (18%)	3 (16%)	1 (7.7%)	10 (29%)	2 (25%)	2 (40%)	1 (17%)	29 (22%)	
Non-smoker	197 (67%)	48 (61%)	13 (68%)	11 (85%)	20 (57%)	4 (50%)	3 (60%)	4 (67%)	94 (73%)	
**Histopathology**										<0.001†
Adenosarcoma	31 (7.6%)	5 (5.7%)	6 (24%)	4 (27%)	7 (15%)	2 (12%)	0 (0%)	2 (6.1%)	5 (3.1%)	
ESS-HG	40 (9.9%)	19 (22%)	1 (4.0%)	1 (6.7%)	2 (4.3%)	2 (12%)	5 (26%)	0 (0%)	10 (6.1%)	
ESS-LG	62 (15%)	14 (16%)	6 (24%)	2 (13%)	2 (4.3%)	4 (24%)	0 (0%)	4 (12%)	30 (18%)	
LMS	221 (54%)	46 (52%)	7 (28%)	4 (27%)	33 (72%)	7 (41%)	12 (63%)	13 (39%)	99 (61%)	
Other	6 (1.5%)	0 (0%)	2 (8.0%)	1 (6.7%)	1 (2.2%)	1 (5.9%)	0 (0%)	0 (0%)	1 (0.6%)	
Sarcoma NOS	46 (11%)	4 (4.5%)	3 (12%)	3 (20%)	1 (2.2%)	1 (5.9%)	2 (11%)	14 (42%)	18 (11%)	
**FIGO Stage**										0.198†
I	212 (58%)	51 (58%)	15 (65%)	7 (47%)	12 (50%)	10 (63%)	11 (92%)	20 (77%)	86 (53%)	
II	49 (13%)	11 (13%)	1 (4.3%)	3 (20%)	3 (13%)	5 (31%)	1 (8.3%)	3 (12%)	22 (14%)	
III	33 (9.0%)	7 (8.0%)	1 (4.3%)	0 (0%)	4 (17%)	0 (0%)	0 (0%)	2 (7.7%)	19 (12%)	
IVA	6 (1.6%)	3 (3.4%)	1 (4.3%)	0 (0%)	1 (4.2%)	0 (0%)	0 (0%)	0 (0%)	1 (0.6%)	
IVB	66 (18%)	16 (18%)	5 (22%)	5 (33%)	4 (17%)	1 (6.3%)	0 (0%)	1 (3.8%)	34 (21%)	

Supplementary Table 1: Baseline demographics and clinical data. Abbreviations: ESS-HG = high grade endometrial stromal sarcoma; ESS-LG = low grade endometrial stromal sarcoma; LMS = leiomyosarcoma; FIGO = The International Federation of Gynaecology and Obstetrics, IQR = interquartile range. *Kruskal-Wallis test; † Chi-squared test

Supplementary Table 2: Survival by stage and tissue diagnosis. Hazard ratio (HR) for survival at indicated time point (95% interval).

	12 Months	24 Months	60 Months	Median Survival (95 % CI)
**Overall**	0.76 (0.72, 0.81)	0.60 (0.56, 0.65)	0.41 (0.37, 0.47)	37 (29, 50)
**FIGO stage**				
I	0.90 (0.86, 0.94)	0.80 (0.74, 0.85)	0.59 (0.53, 0.66)	105 (73, -)
II	0.76 (0.64, 0.89)	0.57 (0.45, 0.73)	0.37 (0.26, 0.54)	33 (19, 72)
III	0.56 (0.42, 0.76)	0.44 (0.30, 0.65)	0.34 (0.21, 0.56)	19 (11, 102)
IVA	0.67 (0.38, 1.0)	0.33 (0.11, 1.0)	- (-, -)	15 (7.9, -)
IVB	0.55 (0.45, 0.69)	0.28 (0.19, 0.41)	0.09 (0.04, 0.20)	14 (10, 18)
**Histopathological subtype**				
Adenosarcoma	0.83 (0.71, 0.98)	0.70 (0.55, 0.88)	0.54 (0.39, 0.77)	76 (34, -)
ESS-HG	0.59 (0.45, 0.77)	0.46 (0.32, 0.64)	0.29 (0.17, 0.48)	18 (9.2, 102)
ESS-LG	0.97 (0.92, 1.0)	0.95 (0.90, 1.0)	0.87 (0.79, 0.96)	- (-, -)
LMS	0.78 (0.72, 0.84)	0.58 (0.52, 0.65)	0.32 (0.26, 0.39)	30 (25, 40)
Other	0.50 (0.22, 1.0)	0.50 (0.22, 1.0)	0.50 (0.22, 1.0)	50 (8.1, -)
Sarcoma NOS	0.54 (0.42, 0.71)	0.32 (0.21, 0.49)	0.25 (0.15, 0.42)	13 (9.2, 22)
