# Supplementary material for: TOURISM study (Treatment Outcomes in UteRIne SarcoMa): a 10-year retrospective evaluation of practice in the UK
Source: BMJ Open. 2024 Dec 26;14(12):e094838. doi: 10.1136/bmjopen-2024-094838 (PMC11683892; doi:10.1136/bmjopen-2024-094838)
Supplement: online supplemental file 2 [file bmjopen-14-12-s002.docx]

NOTCH Protocol

| Title of the Project |
| --- |
| A retrospective evaluation of treatment and outcomes for uterine sarcoma in the United Kingdom 2008 -2017: the TOURISM study (Treatment Outcomes in UteRIne SarcoMa) |
| Personnel Involved |
| The project with be co-ordinated by the team based at the Edinburgh Cancer Centre, Western General Hospital, Edinburgh. The registrar team will be led Dr Karen Mactier (Clinical Oncology) and Dr Karin Purshouse (Medical Oncology). Senior input and advice will be provided by Dr Alison Stillie (Consultant Clinical Oncologist). Expert opinion has also been sought from Dr Scott Fegan (Consultant Gynaecologist), Dr Siobhan McLaughlin (Consultant Radiologist), Dr Helen Creedon (Consultant Medical Oncologist) and Dr Anca Oniscu, Consultant Pathologist) and Clare Forrester (Clinical Nurse Specialist). Patient input has been sought *by proxy* through discussion with a  Sarcoma UK patient advisor.  The project will be commenced initially commenced at two test centres in Scotland. The cancer centres involved will be The Tayside Cancer Centre at Ninewells Hospital (Dr Mark Baxter) and the Edinburgh Cancer Centre (Dr Karen Mactier, Dr Karin Purshouse). It is hoped that after initial set-up the project will be expanded to include other cancer centres throughout the UK via the National Oncology Trainees Collaborative for Healthcare Research (NOTCH) network. |
| Background |
| Uterine sarcomas constitute approximately only 3-7% of uterine malignancies. [**Mbatani**]The main histological subtypes are leiomyosarcomas (67%), which derive from smooth muscle, and endometrial stromal tumours (17-25%), which originate from supporting connective tissue structures, adenosarcomas (5-10%), derived from mixed glandular and stromal types and undifferentiated sarcomas (8-17%)**[Mbatani, D’Angelo].**However, in common with sarcomas in other anatomical sites, a large number of rarer variants can occur including rhabomyosarcoma and giant cell sarcomas**[Abeler, Moinfar].**Carcinosarcomas were previously described in the literature but are now felt to be of epithelial origin and are therefore better characterised and managed as poorly-differentiated adenocarcinomas.**[Amant 2005, McCluggage]**  Sarcomas account for a disproportionately high proportion of uterine cancer deaths in contrast to carcinomas despite their comparative rarity**[De’Angelo, Francis].**However, there remains a wide variation in survival depending on histological sub-type. Analysis from the German Cancer Registry of 937 patients diagnosed between 2009 and 2013 showed five year survival for stage I and II tumours was 53.0% for leiomyosarcoma and 97.2% for low-grade endometrial stromal sarcoma **[Pietzner].**Increasing age and black racial background are also known to adversely affect survival **[Hosh.]**  Most women present via the gynaecologists with symptoms of pelvic pain, bleeding and/or a tumour mass**;** diganosis can be an incidental finding following leiomyoma (fibroid) morcellation surgery **[Trope]**. Consequently many women are initially managed via the gynaecology-oncology rather than sarcoma multi-disciplinary team (MDT). A survey by Sarcoma UK in 2014 highlighted the potential anxiety that can be provoked when patients do not feel they are being managed by the correct clinical team **[Sarcoma UK]**.  The cornerstone of treatment of early stage uterine sarcoma remains surgery. **[Amanti]**Adjuvant treatment of early disease is hampered by a lack of good trial evidence. Radiotherapy can be used with the aim of increasing rates of post-operative local control or to palliate symptoms deaths but does not improve overall survival **[Reed , Livi].** Trials of systemic anticancer agents, including cytotoxic agents and anti-oestrogen or anti-progesterone agents have been undertaken. In common with the radiation trials, they have largely been hampered by small numbers and have failed to demonstrate significant survival benefits**[Desar].**  In keeping with other soft-tissue sarcomas, doxorubicin-based chemotherapy regimens are the treatment of choice in the metastatic setting **[Judson].** However, restrospective analysis from 269 patients treated in the EORTC-STBSG failed to demonstrate a significant treatment benefit from antracycline, ifosfomide, cyclophospamide or combination regimes in this patient group where median overall survival was 10.4 months. **[Ray-Coquart].** Aromatase inhibitors have been used with some success in endometrial stromal tumours [**Thanopoulou**]. More recently targeted agents have been trialled. The VEGF-inhibitor Pazopanib demonstrated a modest benefit whereas and the anti-PDGF-R α monoclonoclonal antibody Olaratumab has now been shown not to improve outcomes in addition to Doxorobicin**[Benson, Tap].**  In summary, uterine sarcomas are a relatively rare, poor-prognosis tumour with limited non-surgical treatment options. Good-quality data on treatments received in clinic by non-trial populations are lacking. |
| What is already known on this topic? |
| Uterine sarcomas are a rare, diverse group of cancers that carries often carries a poor prognosis and significant symptom burden. Good-quality, multi-centre information on treatment and patient outcomes is lacking in the literature. Results are often extrapolated from trials including soft-tissue sarcomas from other anatomical sites. Consequently, it can be difficult for the treating clinician to be confident in selecting the correct treatment plan for their patient.Patient advocacy groups suggest that patients do not always receive review by a sarcoma specialist team. |
| **What will this study add?** |
| The primary aim of the project is to provide an up-to-date assessment of current treatment modalities of the most common-subtypes of uterine sarcoma in UK practise, namely   - surgery - radiotherapy - systemic anti-cancer therapy (SACT)   Secondary aims   - provide detailed demographic detail on patients treated - Describe the survival outcomes for the cohort - assess for regional variations in practise - determine the proportion of patients treated by an oncologist with a specialist interest in sarcoma   It is hoped this study would provide high quality relevant data which could be utilised for future service development. |
| \| Aims \| \| --- \| \| This project would aim to give a descriptive oversight of the presentation and management uterine sarcomas within the United Kingdom over the past 10 years.  The objective is to produce a comprehensive report of current oncological practice regarding these relatively rare tumours that would have the potential to highlight disparities across the country and inform service development. \| \| Study Design \| \| This will be a retrospective descriptive study design.  When defining the period of interest a balance must be struck between choosing a recent time period to reflect contemporary practice and obtaining sufficient patient numbers for this relatively rare group of malignancies. The period of interest will be 1^st^January 2008 to 31^st^December 2017. All patients diagnosed with a uterine sarcoma within these time limits will be included. Patient follow-up will be censored at December 2020 to prevent inconsistencies across different sites.  Each centre will identify patients via in local Cancer Advisory Network Cancer Registries. The World Health Organization and International Statistical Classification of Diseases and Related Health Problems version 10 (WHO ICD-10 Version:2016) and International Classification of Diseases for Oncology, 3rd Edition (ICD-O-3)diagnostic criteria will be used to define the search for patients with the appropriate histological diagnoses.  Relevant clinical information will be collected via review of the patients’ case notes. All patients identified will have a standard set of denominator demographics collected as correct at the time of diagnosis. Information will be stored on the study Data Collection Tool which will include pre-defined fields.  The Union for International Cancer control (UICC) TNM Classification of Malignant Tumours, 7th Edition, and the International Federation of Gynaecology and Obstetrics (FIGO) 2009 staging protocols would have been the most widely used during the period of interest and therefore will be used in this study. It is important to note that adenosarcomas have a different staging system to the other histopathological sub-types. \| \| **Patient Identification** \| \| Inclusion Criteria   - Patients must be over 18 years old. - Histopathologically confirmed diagnosis of primary uterine sarcoma between 1^st^ January 2008 and 31^st^ December 2017:   - Diagnosis of primary uterine malignancy, i.e. ICD-10 code     - C53 Malignant neoplasm of the cervix uteri*or*     - C54 Malignant neoplasm of the corpus uteri*or*     - C55 Malignant neoplasm of uterus, part unspecified   - Histopathological confirmed diagnosis of sarcoma, i.e. ICD-O-3 code     - 8930/3 endometrial, NOS (C54.1) *or*     - 8930/3 Endometrial stromal NOS (C54.1) *or*     - 8930/3 high grade (C54.1) *or*     - 8931/3 low grade (C54.1) *or*     - 8890/3 Leiomyosarcoma, NOS *or*     - 8800/3 Sarcoma NOS *or*     - 8933/3 Adenosarcoma *or*     - 9260/3 Ewing sarcoma - Patients must have accessible medical records for review   Exclusion Criteria   - Carcinosarcomas/ mixed Mullerian tumours - Gynaecological sarcoma arising from non-uterine structures, i.e. adnexae, ovary, vagina, vulva - Patients with cancer from extra-uterine primary sites that have metastasised to the uterus.   All patients will be designated a study ID by the assessing centre.  USXXXXY where X is the patient numbers and Y is the centre code  Centre codes:  Edinburgh – ED  Dundee – DU  Glasgow – GL  Aberdeen – AB  I.e. the first patient reviewed in Edinburgh would be US0001ED  Patient identifiable information will be stored in password-protected Microsoft Excel files in each centre on NHS servers. No patient identifiable information will be transferred to the lead centre. Pseudo-anonymised data (i.e. with study IDs) will be emailed using NHS emails to NHS Lothian and transferred to a password-protected study Excel file. \| \| Data Fields \| \| *General demographics*  Date of diagnosis  Date of referral to secondary care  Age at diagnosis of uterine sarcoma  Ethnicity (Appendix A)  Height  Weight  Body Mass Index  Menopausal status at diagnosis (Appendix B)  Parity (Nulliparous/ parous)  Smoking status (Appendix C)  Living status (Alive/Dead)  Date of Death  *Diagnostic information*  Anatomical sub-site (Appendix D)  Histopathological subtype (Appendix E)  FIGOstage at diagnosis (Appendix F)  TNM Primary tumour staging (Appendix G)  TNM Regional lymph node staging (Appendix H)  TNM Metastasis staging (Appendix I)  *Treatment details*  Surgical treatment (Appendix J)  Radiotherapy (Appendix K)  Systemic therapy (Appendix L)  *Clinicians involved in patient care*  Gynaecologist (Y/N)  Oncologist with specialist interest in gynaecological cancer (Y/N)  Oncologist with a specialist interest in sarcoma (Y/N) \| \| **Statistical Analysis** \| \| Descriptive analysis.  Odd ratios and multivariate analysis may be used to test the data should variations be found.  3,876 patients were diagnosed with uterine cancer in Scotland between 2014 and 2018 which translates to an estimated uterine sarcoma incidence of 23 and 55 cases in Scotland annually (assuming between 3% and 7% of uterine cancers are sarcomas). \| \| **Ethics and NHS Approvals** \| \| A submission for Research and Development Ethics Committee approval will be made via the Integrated Research Application System (Project ID 292971). An application to the NHS Lothian Caldicott Guardian’s office will be made to store the anonymised data centrally. Separate applications to the Caldicott Guardian of each participating NHS Health Boards/Trust will be needed in order to transfer anonymised data outwith the NHS organisation to NHS Lothian. \| \| \| **Projected Timeline** \| \| --- \| \| Jan 2021 – submission for Caldicott and R&D approval (Edinburgh)  March 2021 – recruitment of personnel at participating sites  April 2021 – Caldicott applications at participating sites  May - August 2021 data collection  Sept – Oct 2021 – Analysis of data centrally (Edinburgh)  Nov 2021 – Jan 2022 manuscript preparation for submission for publication \| \|  \| \| \| **References** \| \| [Reed] Reed NS, Mangioni C, Malmström H, Scarfone G, Poveda A, Pecorelli S, Tateo S, Franchi M, Jobsen JJ, Coens C, Teodorovic I, Vergote I, Vermorken JB; European Organisation for Research and Treatment of Cancer Gynaecological Cancer Group. Phase III randomised study to evaluate the role of adjuvant pelvic radiotherapy in the treatment of uterine sarcomas stages I and II: an European Organisation for Research and Treatment of Cancer Gynaecological Cancer Group Study (protocol 55874). Eur J Cancer. 2008 Apr;44(6):808-18. doi: 10.1016/j.ejca.2008.01.019. Epub 2008 Apr 2. Erratum in: Eur J Cancer. 2008 Jul;44(11):1612. PMID: 18378136.[D’angelo] D'Angelo E, Prat J. Uterine sarcomas: a review. Gynecol Oncol. 2010 Jan;116(1):131-9. doi: 10.1016/j.ygyno.2009.09.023. Epub 2009 Oct 23. PMID: 19853898. [Francis]Francis M, Dennis NL, Hirschowitz L, Grimer R, Poole J, Lawrence G, Nordin A. Incidence and survival of gynecologic sarcomas in England. Int J Gynecol Cancer. 2015 Jun;25(5):850-7. doi: 10.1097/IGC.0000000000000443. PMID: 25853381.    [Mbatani]Mbatani N, Olawaiye AB, Prat J. Uterine sarcomas. Int J Gynaecol Obstet. 2018 Oct;143 Suppl 2:51-58. doi: 10.1002/ijgo.12613. PMID: 30306577.  [Amant] Amant F, Coosemans A, Debiec-Rychter M, Timmerman D, Vergote I. Clinical management of uterine sarcomas. Lancet Oncol. 2009 Dec;10(12):1188-98. doi: 10.1016/S1470-2045(09)70226-8. PMID: 19959075.  [Abeler] Abeler VM, Røyne O, Thoresen S, Danielsen HE, Nesland JM, Kristensen GB. Uterine sarcomas in Norway. A histopathological and prognostic survey of a total population from 1970 to 2000 including 419 patients. Histopathology. 2009 Feb;54(3):355-64. doi: 10.1111/j.1365-2559.2009.03231.x. PMID: 19236512.  [Amant] Amant F, Moerman P, Neven P, Timmerman D, Van Limbergen E, Vergote I. Endometrial cancer. Lancet. 2005 Aug 6-12;366(9484):491-505. doi: 10.1016/S0140-6736(05)67063-8. PMID: 16084259.  (McCluggage] McCluggage WG. Uterine carcinosarcomas (malignant mixed Mullerian tumors) are metaplastic carcinomas. Int J Gynecol Cancer. 2002 Nov-Dec;12(6):687-90. doi: 10.1046/j.1525-1438.2002.01151.x. PMID: 12445244.  [Pietzner]Pietzner K, Buttmann-Schweiger N, Sehouli J, Kraywinkel K. Incidence Patterns and Survival of Gynecological Sarcoma in Germany: Analysis of Population-Based Cancer Registry Data on 1066 Women. Int J Gynecol Cancer. 2018 Jan;28(1):134-138. doi: 10.1097/IGC.0000000000001128. PMID: 29040191.  [Moinfar] Moinfar F, Azodi M, Tavassoli FA. Uterine sarcomas. Pathology. 2007 Feb;39(1):55-71. doi: 10.1080/00313020601136146. PMID: 17365823.  [Trope] Tropé CG, Abeler VM, Kristensen GB. Diagnosis and treatment of sarcoma of the uterus. A review. Acta Oncol. 2012 Jul;51(6):694-705. doi: 10.3109/0284186X.2012.689111. PMID: 22793037.  [Thanopoulou] Thanopoulou E, Aleksic A, Thway K, Khabra K, Judson I. Hormonal treatments in metastatic endometrial stromal sarcomas: the 10-year experience of the sarcoma unit of Royal Marsden Hospital. Clin Sarcoma Res. 2015 Mar 15;5:8. doi: 10.1186/s13569-015-0024-0. PMID: 25810898; PMCID: PMC4373094.  [Benson] Benson C, Ray-Coquard I, Sleijfer S, Litière S, Blay JY, Le Cesne A, Papai Z, Judson I, Schöffski P, Chawla S, Gil T, Piperno-Neumann S, Marréaud S, Dewji MR, van der Graaf WTA. Outcome of uterine sarcoma patients treated with pazopanib: A retrospective analysis based on two European Organisation for Research and Treatment of Cancer (EORTC) Soft Tissue and Bone Sarcoma Group (STBSG) clinical trials 62043 and 62072. Gynecol Oncol. 2016 Jul;142(1):89-94. doi: 10.1016/j.ygyno.2016.03.024. Epub 2016 Apr 29. PMID: 27012429.  [Tap] Tap WD, Wagner AJ, Schöffski P, Martin-Broto J, Krarup-Hansen A, Ganjoo KN, Yen CC, Abdul Razak AR, Spira A, Kawai A, Le Cesne A, Van Tine BA, Naito Y, Park SH, Fedenko A, Pápai Z, Soldatenkova V, Shahir A, Mo G, Wright J, Jones RL; ANNOUNCE Investigators. Effect of Doxorubicin Plus Olaratumab vs Doxorubicin Plus Placebo on Survival in Patients With Advanced Soft Tissue Sarcomas: The ANNOUNCE Randomized Clinical Trial. JAMA. 2020 Apr 7;323(13):1266-1276. doi: 10.1001/jama.2020.1707. PMID: 32259228; PMCID: PMC7139275.  [Ray-Coquard] Ray-Coquard I, Rizzo E, Blay JY, Casali P, Judson I, Hansen AK, Lindner LH, Dei Tos AP, Gelderblom H, Marreaud S, Litière S, Rutkowski P, Hohenberger P, Gronchi A, van der Graaf WT. Impact of chemotherapy in uterine sarcoma (UtS): review of 13 clinical trials from the EORTC Soft Tissue and Bone Sarcoma Group (STBSG) involving advanced/metastatic UtS compared to other soft tissue sarcoma (STS) patients treated with first line chemotherapy. Gynecol Oncol. 2016 Jul;142(1):95-101. doi: 10.1016/j.ygyno.2016.05.016. Epub 2016 May 24. PMID: 27208537. \| \| Appendices  Appendix A  Ethnicity   1. White 2. White Irish 3. Other White 4. Mixed: White and Black Caribbean 5. Mixed: White and Black African 6. Mixed: White and Asian 7. Other mixed background 8. Indian 9. Pakistani 10. Bangladeshi 11. Other Asian background 12. Caribbean 13. African 14. Other Black background 15. Chinese 16. Other ethnic group 17. Unknown   Appendix B  Menopausal Status   1. Pre-menopausal 2. Peri-menopausal 3. Post-menopausal 4. Unknown   Appendix C  Smoking Status   1. Current smoker 2. Ex-smoker 3. Non-smoker 4. Unknown   Appendix D  Anatomical sub-site   1. Cervix uteri 2. Corpus uteri 3. Uterus, NOS   Appendix E  Histological subtype   1. Leiomyosarcoma 2. Endometrial stromal sarcoma – high grade 3. Endometrial stromal sarcoma – low grade 4. Adenosarcoma 5. Other subtype 6. Sarcoma, NOS   Appendix F  FIGO staging at diagnosis   1. Stage 0 2. Stage IA^#^ 3. Stage IB^#^ 4. Stage IC^##^ 5. Stage IIA 6. Stage IIB 7. Stage IIIA 8. Stage IIIB 9. Stage IIIC 10. Stage IVA 11. Stage IVB   ^#^Stage IA and IB for adenosarcoma differ from those applied to leiomyosarcoma and endometrial stromal sarcoma  ^##^ Stage IC does not apply for leiomyosarcoma and endometrial stromal sarcoma  Appendix G  TNM Primary tumour staging   1. T0 2. TX 3. T1a Tumour is limited to the endometrium/endocervix without myometrial invasion^#^ 4. T1b Tumour invades less than or equal to 50% (≤50%) total myometrial thickness ^##^ 5. T1c Tumor invades greater than 50% (>50%) total myometrial thickness 6. T2a ( Tumour involves the adnexa) 7. T2b (Tumour involves other pelvic tissue) 8. T3a (Tumour invades abdominal tissues at one site) 9. T3b (Tumour invades abdominal tissues at more than one site) 10. T4( Tumour invades bladder mucosa and/or rectum)   ^#^For Non-adenosarcomas T1a is defined as tumour ≤5cm in greatest dimension  ^##^ For non-adenosarcomas T1b is defined as tumour >5cm in greatest dimension  ^###^ T1c not defined for non-adenosarcomas  Appendix H  TNM Regional lymph node staging   1. No 2. N1^#^ 3. NX   ^#^Regional lymph nodes include the pelvic, obturator, internal iliac (hypogastric), external iliac, common iliac, paraaortic, presacral, and parametrial lymph nodes.  Appendix I  TNM Metastasis staging   1. M0 2. M1 3. MX   Appendix J  Surgical treatment   1. Oncological Total abdominal hysterectomy + bilateral salphino-opherectomy (TAH-BSO) 2. Total hysterectomy 3. Leiomyoma (fibroid) surgery 4. Debulking 5. Biopsy 6. Other surgical procedure 7. No surgery   Appendix K  Medical professionals involved in care^α^   1. Gynaecologist 2. Oncologist with a specialist interest in gynaecological cancer^β^ 3. Oncologist with a specialist interest in sarcoma^β^   α Involvement should be defined as a clinical encounter, whether inpatient or outpatient  β Oncologist will be a core member of the multidisciplinary team meeting for gynae-onc or sarcoma respectively \| |
