# Supplementary material for: TOURISM study (Treatment Outcomes in UteRIne SarcoMa): a 10-year retrospective evaluation of practice in the UK
Source: BMJ Open. 2024 Dec 26;14(12):e094838. doi: 10.1136/bmjopen-2024-094838 (PMC11683892; doi:10.1136/bmjopen-2024-094838)
Supplement: Supplementary file 3 [file bmjopen-2024-094838.reviewer_comments.pdf]

PEER REVIEW HISTORY

BMJ Open publishes all reviews undertaken for accepted manuscripts. Reviewers are asked to complete a checklist review form and are provided with free text boxes to elaborate on their assessment. These free text comments are reproduced below.

ARTICLE DETAILS

Title (Provisional)

The TOURISM study (Treatment Outcomes in UteRIne SarcoMa): A 10-year retrospective evaluation of practice in the United Kingdom

Authors

Mactier, Karen Elizabeth; Baxter, Mark A; Peters, Adam Louis Louis; Fair, Katherine; Hannington, Laura; Robertson, James; Wood, Georgina; Sarwar, Asma; Bishr, Mai; Webb, Rebekah; Al-Zubaidi, Mohammed; Eastlake, Leonie; Lankester, Katherine; McInerney, Samuel; Creedon, Helen; Stillie, Alison; Purshouse, Karin

---

VERSION 1 - REVIEW

**Reviewer**	**1**
**Name**	**Giannini, Andrea**
**Affiliation**	**University of Rome La Sapienza**
**Date**	**02-Nov-2024**
**COI**	

I read with great interest the Manuscript titled " The TOURISM study (Treatment Outcomes in UteRIne SarcoMa): A 10-year retrospective evaluation of practice in the United Kingdom" which falls within the aim of the Journal.

Although the manuscript can be considered already of good quality, I would suggest following recommendations:

- I suggest round of language revision, in order to correct few typos and improve readability;

- Considering topics of this study, it would be interesting to add few lines about the difficult management of uterine sarcoma. In particular authors could consider the risk of facing off of a misdiagnosed sarcoma during the management of these lesions. I would be glad if the authors discuss this important point, referring to PMID: 37348383.

- I suggest reading and adding recent evidence about surgical approaches of this topic, considering surgical considerations, safety and fertility concerns, long-term outcomes and the risk of facing off of a misdiagnosed sarcoma during the management of benign lesions.

Considering results of this study, I suggest reading and adding recent evidence about this topic.

I would be glad if the authors discuss this important point, referring to PMID: 38581885

Because of these reasons, the article should be revised and completed. Considering all these points, I think it could be of interest to the readers and, in my opinion, it deserves the priority to be published after minor revisions.

**Reviewer**	**2**
**Name**	**Teixeira, Julio**
**Affiliation**	**State University of Campinas, Gynecology and Obstetrics**
**Date**	**15-Nov-2024**
**COI**	

Congratulations on the initiative and the effort to provide more information on a topic that is particularly tragic for gynecological oncologists, who are often the first line of care for the majority of these cases. The manuscript is well-written, clear, and presents results that align with the perception of routine care for women with uterine sarcomas.

After carefully evaluating the manuscript and its relevance to clinical practice, I found that two pieces of information were missing:

[1] The parity of the women involved, as a similar study from 2020 with 122 cases (Plentz et al; DOI: 10.3892/mco.2020.2151) reported that 30% had 3-4 births and 30% had 5 or more births.

[2] The signs or symptoms that led to the diagnosis. In the same article by Plentz et al., over 70% of patients presented with genital bleeding. This is crucial information, as gynecologists are likely to encounter these cases.

I believe this information is essential, as it could help raise awareness among gynecologists about the possibility of sarcomas when investigating potential endometrial cancer. Moreover, the understanding that biopsy procedures could exacerbate the already poor prognosis of sarcomas—by potentially promoting the spread and metastasis of the disease—would be a significant addition to the manuscript's clinical relevance.

VERSION 1 - AUTHOR RESPONSE

Reviewer: 1
Dr. Andrea Giannini, University of Rome La Sapienza
Comments to the Author:
I read with great interest the Manuscript titled " The TOURISM study (Treatment Outcomes

in UteRIne SarcoMa): A 10-year retrospective evaluation of practice in the United Kingdom" which falls within the aim of the Journal.

Although the manuscript can be considered already of good quality, I would suggest following recommendations:
- I suggest round of language revision, in order to correct few typos and improve readability;

Please see various corrections throughout the main document

- Considering topics of this study, it would be interesting to add few lines about the difficult management of uterine sarcoma. In particular authors could consider the risk of **facing off of a misdiagnosed sarcoma during the management of these lesions**. I would be glad if the authors discuss this important point, referring to PMID: 37348383.

Reference made to tumour seeding, with particular regards to tumours later diagnosed as stage IVB given our cohort findings within the discussion, with citation to Bogani et al 2016. Please note that PMID: 37348383 has already been cited within the manuscript.

- I suggest reading and adding recent evidence about surgical approaches of this topic, considering **surgical considerations, safety and fertility concerns, long-term outcomes and the risk of facing off of a misdiagnosed sarcoma during the management of benign lesions**. Considering results of this study, I suggest reading and adding recent evidence about this topic.
I would be glad if the authors discuss this important point, referring to PMID: 38581885 (2024)

We read with interest Giannini *et al*.'s meta-analysis PMID: 38581885. This study compares rates of intra-operative, post-operative and long-term complications for laproscopic vs. open myomectomy. The vast majority of patients described therein have benign uterine leiomyomas. Undiagnosed uterine sarcoma in relation to morcellation is discussed briefly in the Discussion with reference to PMID: 38581885 (see above) and two papers by Bogani *et al.*.

Unfortunately, more nuanced information on surgical approach and complications, beyond the information contained in Table 2 of the manuscript, was not collected during this study. Morcellation was only performed in 3 (0.8%) of patients in this study (1x stage I LMS, 1x stage II ESS-LG and 1x stage IVB LMS). We therefore feel that a comprehensive review of the risks inherent to this surgical technique with respect to undiagnosed uterine sarcoma is out with the scope of this paper and our clinical expertise as non-surgical oncologists. I concession to this effect has been added to the Limitations.

Because of these reasons, the article should be revised and completed. Considering all these points, I think it could be of interest to the readers and, in my opinion, it deserves the priority to be published after minor revisions.

Reviewer: 2
Dr. Julio Teixeira, State University of Campinas
Comments to the Author:
Congratulations on the initiative and the effort to provide more information on a topic that is particularly tragic for gynecological oncologists, who are often the first line of care for the

majority of these cases. The manuscript is well-written, clear, and presents results that align with the perception of routine care for women with uterine sarcomas.

After carefully evaluating the manuscript and its relevance to clinical practice, I found that two pieces of information were missing:

[1] **The parity of the women involved**, as a similar study from 2020 with 122 cases (Plentz et al; DOI: 10.3892/mco.2020.2151) reported that 30% had 3-4 births and 30% had 5 or more births.

The median parity has been added to the first paragraph of the Results. Further information on parity in the cohort has been provided below for the reviewers' interest:

Median 2 (IQR 0-3) range (0-9). Mean 1.77 se 0.09 ci 0.169

Nulliparous 26.6%, single parity 16.0%, multiparity 51.4%, grandmultiparity 6.0%

[2] **The signs or symptoms that led to the diagnosis.** In the same article by Plentz et al., over 70% of patients presented with genital bleeding. This is crucial information, as gynecologists are likely to encounter these cases.
I believe this information is essential, as it could help raise awareness among gynecologists about the possibility of sarcomas when investigating potential endometrial cancer. Moreover, **the understanding that biopsy procedures could exacerbate the already poor prognosis** of sarcomas—by potentially promoting the spread and metastasis of the disease—would be a significant addition to the manuscript's clinical relevance.

Unfortunately, as information was collected retrospectively, with case identification via cancer registries and review of primarily oncological, rather than surgical notes, information on presenting complaint leading to diagnosis was sparse and therefore of insufficient quality for inclusion in the manuscript. The authors appreciate the importance of the information regarding presenting complaint, and have included the findings of Plentz *et al.* in the Introduction.

We appreciate there is ongoing discussion regarding risks of tumour seeding with biopsy. Current UK guidelines for soft tissue and uterine sarcomas recommend triple assessment (history, imaging, biopsy) in a specialist centre supervised by an expert clinician to minimise the risk of tumour seeding. UK guidelines for the management of soft tissue sarcomas - PMC British-Gynaecological-Cancer-Society-v13-for-website-with-figure1.pdf Given the manuscript focusses primarily on treatment strategies for uterine sarcoma, and histopathological diagnosis is of upmost importance for prognostication and subsequent management, we feel a robust discussion of the potential risks of biopsy are out with the scope of this article and may appear to contradict national guidance.
